# Physicians' Perspective on Vaccine-Hesitancy at the Beginning of Israel's COVID-19 Vaccination Campaign and Public's Perceptions of Physicians' Knowledge When Recommending the Vaccine to Their Patients: A Cross-Sectional Study

**DOI:** 10.3389/fpubh.2022.855468

**Published:** 2022-03-10

**Authors:** Anat Gesser-Edelsburg, Hiba Badarna Keywan

**Affiliations:** School of Public Health and the Health and Risk Communication Lab, University of Haifa, Haifa, Israel

**Keywords:** COVID-19, vaccine hesitancy, HCWs, physicians' perspective, public's perceptions, vaccination campaign, Israel, cross-sectional study

## Abstract

Because public healthcare workers (HCWs) are at the forefront of the battle against COVID-19, they must be able to provide vaccination information to their patients and respond to their anxieties and concerns. This research objectives were to (1) examine physicians' perceptions of how they received information about the Pfizer COVID-19 vaccine, their attitudes toward hesitant colleagues, and their own knowledge and self-efficacy in communicating information to their patients, and (2) to examine the public's perceptions of physicians' knowledge when recommending the COVID-19 vaccine to their patients. At the beginning of the vaccination campaign, a survey examined the attitudes of physicians in the Israeli public healthcare system (*n* = 295) regarding the Pfizer vaccine. In addition, the attitudes of a representative sample (*n* = 500) of the Israeli adult population (age 18+) were examined through interviews. Most of the participating physicians (81%) reported they had already been vaccinated or intended to be vaccinated. When asked about their reasons for vaccine hesitancy, 27% cited concerns about long-term side effects and doubts about the vaccine's effectiveness in preventing contagion. They cited system pressure and departmental norms as explanations for their eventual compliance. Moreover, they saw the system as less tolerant of hesitant physicians, while they themselves tend to be more tolerant. The results of the survey of the public showed that mostly young people (under 44) who tend to be critical believe that physicians do not have sufficient knowledge to make recommendations about the COVID-19 vaccine. The findings indicate that the health system should employ complete transparency in conveying the advantages and disadvantages of the COVID-19 vaccine to physicians. The system should be more tolerant of physicians' worries and concerns and grant legitimacy to their reservations and misgivings. Moreover, medical studies should reinforce physicians' immunological knowledge regarding vaccinations so they can help their patients make informed decisions.

## Introduction

Public healthcare workers (HCWs) are a primary source of information about vaccinations (1, 2). In their position at the forefront of the battle against COVID-19, HCWs must be able to provide information to their patients and respond to their anxieties and concerns (3). Indeed, for many subgroups in the population, HCWs are the main source of information about vaccinations (4).

Research shows that HCWs play a crucial role in their patients' vaccination compliance (5, 6). In today's new media world information is not exclusive and the public draws upon various sources of information (7). Hence, it is even more important for HCWs to have up-to-date and comprehensive information (8) in order to serve as a reliable source for their patients (9).

Moreover, research on vaccinations over the years as well as studies conducted during the COVID-19 crisis has pointed to three main barriers that prevent HCWs from fully meeting these needs. First, many HCWs themselves have a low level of compliance with vaccinations they should be receiving. Their vaccination barriers also resemble those of the general public and include concerns about side effects and vaccination novelty, as well as a lack of faith in the vaccine's efficacy and disease severity (10–13). Studies indicate that HCWs who choose to get vaccinated (14–17) encode the same epidemiological data differently than those who do not get vaccinated (18, 19).

Second, many studies focus on physicians' and nurses knowledge of the procedural aspects of giving vaccinations, such as vaccination timetables (20), their knowledge of official recommendations (21–23), and on their ability to recommend and convince their patients rather than on the depth of their knowledge. In addition, a large portion of research studies focus on HCWs' viewpoints on vaccinations, particularly those that are the topic of scientific controversy, such as the HPV vaccination (24, 25). The goal of these studies is to attempt to understand the difficulties experienced by HCWs in conveying vaccination information and to help them communicate the importance of vaccinations to the public (26–30).

These studies are in line with the approach according to which health organizations and health authorities view health professionals as representatives of the establishment whose role is to mediate between the organization and the public (31).

Similarly, most studies conducted during the COVID-19 pandemic focused on physicians' viewpoints regarding the COVID-19 vaccination. A substantial portion of these studies began before the vaccination campaign and sought to assess HCWs' intentions regarding vaccination (32–35). Some of them found that at the beginning of the campaign HCWs had anxieties and concerns about the vaccine's safety and effectiveness (32, 36). Others, among them a study conducted in China, indicated that prior to the beginning of the vaccination campaign HCWs' response was high, but this response decreased in view of their anxieties and their concerns about side effects (37).

Some studies on the COVID-19 vaccine also compared physicians' viewpoints regarding the vaccine to those of nurses (38). Most pointed to a higher response rate among physicians than among nurses (39) and indicated that gender is an influential factor, such that men tend to be more compliant than women (40). In conclusion, the studies deal with viewpoints toward vaccines of health workers and their barriers to getting vaccinated and not with their immunological knowledge about vaccines.

Third, medical studies are marked by gaps and inconsistencies with respect to vaccinations. Many studies reveal gaps in medical students' knowledge regarding their self-efficacy in communicating information about vaccinations (41). A specific work package in the EU project on Vaccine Safety, Attitudes, Training and Communication (VACSATC) (6) focused on possible improvements in pre-service training of future healthcare workers.

In December 2020 the Israeli government announced it was planning to import the Pfizer pharmaceutical company's vaccine against the coronavirus. The Food and Drug Administration (FDA) granted the Pfizer vaccine emergency authorization as an experimental drug (IND) until February 2023, when the clinical trials were scheduled to end. Shortly thereafter, Israel signed a contract with Pfizer, that parts it were concealed from the public (42), and thus became an experimental laboratory for Pfizer (43) and the world, according to Pfizer CEO Albert Burla in an interview with NBC. In this interview, Burla expressed the hope that within a short period of one or two months we would know whether the company's vaccine not only helped protect people from contracting the disease but also stopped contagion. Within less than a month (44), the Israel Ministry of Health recommended vaccinating medical personnel and at-risk populations, and subsequently recommended vaccinating the entire population over the age of 16. At the time of writing this paper, the MOH's advisory board has also recommended vaccinating children ages 5–11 (45, 46).

Since the beginning of the Israeli vaccination campaign, accumulating research has pointed to a number of facts: The vaccine was developed rapidly and uses a technology that has never been used before (47–49). The Pfizer vaccine does not prevent contagion (50) and does not prevent the virus from spreading in the population. On the other hand, evidence from some studies found that the vaccine prevents serious illness in risk groups, reduces chances of developing Long Covid Syndrome (51) and SARS, and reduces serious complications, hospitalization and death (52, 53).

Experts differ regarding the effectiveness of the vaccine and the need to give it to young adults ages 16–40 without underlying conditions, let alone children. Those who oppose the government's sweeping recommendations to vaccinate the entire population cite the absence of long-term and thorough research to follow up the vaccine's side effects and express concerns about side effects that have already appeared (54), such as myocarditis in young people (55) and coagulation problems (56). These opponents argue against universal vaccination, stating that at-risk populations and people with underlying conditions should be vaccinated, whereas the rest of the population should be given the right to make autonomous decisions. They should not be subject to direct or indirect coercion (e.g., by the Green Pass that confers privileges on those who have been vaccinated) (57). They also contend that emergency vaccination of children should not be recommended (58), both because COVID-19 is not dangerous for children and due to concerns about vaccine safety (54).

In contrast, those in favor of universal vaccination claim that both cost-benefit calculations and public health perspectives indicate that the vaccine is safe and effective (59) and that its advantages outweigh its disadvantages (60, 61).

The debate between various experts concerning what public policies should be adopted during the COVID-19 pandemic (e.g., lockdowns, wearing masks, testing for symptoms and vaccinations) has exposed the depth of the conflict (62) between experts representing government ministries in Israel and experts who are critical of government policy. Today in Israel we see evidence that the discourse on vaccination policy in the medical community is being silenced, in particular the deliberate silencing of physicians who have spoken out against the establishment (63).

In view of the above discussion, research studies that examine physicians' attitudes regarding the COVID-19 vaccine are of major importance. As noted, until now most studies on vaccinations, and specifically on the COVID-19 vaccination, have focused on physicians' positions on the vaccine and their reports on vaccine compliance. Very few studies have focused on whether physicians believe they have the immunological knowledge and ability to become familiar with the vaccine and recommend it to their patients, nor have they examined the public's perception of physicians' knowledge and ability to communicate information about a new vaccine during a pandemic.

This health communication study conducted at the beginning of the vaccination campaign in Israel attempts to investigate these issues. This study not only examines physicians' viewpoints regarding the vaccine and their own reported vaccination compliance. Rather, it also focuses on their dilemmas about being vaccinated and their perceptions of vaccine-hesitant colleagues. It also examines the nature of physicians' self-efficacy in recommending the vaccine to their patients, as well as the public's perceptions of physicians' ability to make such recommendations. In addition, the study examines the public's perceptions of physicians' knowledge when recommending the COVID-19 vaccine to their patients.

## Methods

### Study Design and Settings

We conducted a cross-sectional study among physicians in the Israeli public healthcare system on their attitudes toward the Pfizer vaccine, and among the Israeli public regarding perceptions of physicians' knowledge when recommending the COVID-19 vaccine to their patients, at the beginning of the vaccination campaign during 2021.

### Study Population, Sample Size and Sampling

We recruited 295 physicians from different areas of specialization working in the community and in hospitals who continued to see patients during the pandemic. The study did not include retired physicians, physicians on paid leave or maternity leave during the pandemic, physicians in administrative positions at health maintenance organizations (HMO) or at the Health Ministry, or physicians who do not work directly with patients (e.g., pathologists, hospital directors, physicians who work as controllers and on committees). The study among the Israeli public entailed interviewing a representative sample (*n* = 500) of the Israeli adult population (age 18+).

The physicians study sample size was calculated relative to the degree to which the healthcare system tolerates hesitant or anti-vaccine physicians and the degree to which physicians themselves believe hesitant physicians should be tolerated. It was based on the assumption of a gap of one half point between tolerance and non-tolerance. Hence we set the following parameters for the calculation: (a) an average gap of half a point between the two attitudes; (b) a standard deviation of 1.5 points; (c) a negative correlation of −0.30 between the two attitudes (the more physicians believe the system must tolerate anti-vaccine physicians, the less they see the system as tolerant); (d) 90% power; (e) 5% significance level. This calculation indicated that a sample of at least 248 respondents was needed. Since previous studies in the medical literature about attitudes toward vaccines showed that a sample of 295 physicians could provide sufficient information, we decided to increase the sample to 295 respondents. The public study sample size was set to 500 participants, a common practice representative sample size of the Israeli adult population (age 18+).

The study among the physicians was a deliberate sample that sought to reach a population of physicians from various areas of specialization working in different sectors of the Israeli public health system during the COVID-19 pandemic, which broke out in early 2020. The interviewees in the study among the public were sampled from iPanel, the largest internet panel in Israel.

### Study Tools and Data Collection Methods

For the the physicians study we have constructed a questionnaire that included 27 questions. After constructing the questionnaire, we created indexes by calculating the Cronbach's alpha value for items that appeared to be related. The first set of questions referred to sociodemographic and personal information, including age, gender, area of specialization, sector (community vs. hospital) and ethnicity. The second part of the questionnaire included questions regarding respondents' vaccination intentions and their pro and con vaccination considerations. For example: If you are hesitant about the vaccination/refuse to be vaccinated, select the motives for your attitude. The third part of the questionnaire refers to physicians' self-efficacy in recommending the vaccination. For example: To what extent do you agree with the following statement: Physicians have sufficient information to recommend the COVID-19 (5-point Likert scale). The statements were collected and defined as independent variables according to topic. For instance, statements about physicians' knowledge about the COVID-19 vaccine were collected as a single index, with a Cronbach's alpha of 0.743. The fourth part of the questionnaire referred to respondents' assessments of the government's policy and the media strategy it used to communicate information.

The fifth part of the questionnaire examined tolerance for hesitant physicians in the healthcare system and among other physicians. Two questions (Do you feel the administration of the institution where you work allows physicians to express doubts or reservations about the vaccine? Does the system tolerate physicians who publicly criticize the vaccine in the media?) were collected under a single index (Cronbach's alpha = 0.553).

The questionnaire ended with an open question in which respondents were asked to indicate what additional information they would like to have about the Pfizer vaccine.

For the public study, we used a questionnaire examining public attitudes on a variety of topics related to the COVID-19 crisis (64, 65). We focused on a question that examined the public's perceptions of physicians' knowledge: To what extent do you believe the following statement is correct: Physicians in the public health system do not have sufficient information about the COVID-19 vaccine and therefore cannot advise their patients (4-point scale). The questionnaire also examined socio-demographic and personal attributes relevant to the issue of critical thinking, such as conservatism, locus of control and tendency to be critical (Supplementary Material).

The data for the physicians study was collected by distributing an anonymous questionnaire via Google docs during January 2021.The questionnaire was distributed on social networks using two main channels: (1) WhatsApp application: The questionnaire was distributed to dedicated WhatsApp groups of physicians as well as by asking physicians to distribute the questionnaire to colleagues in order to reach maximum exposure (snowball sampling), and (2) Facebook: The questionnaire was posted on Facebook groups of Israeli physicians, after asking for permission from the group administrators.

The data for the public study was collected during the second week of January 2021 via telephone or the internet. Among Arabs and ultra-Orthodox participants, most of those over the age of 55 were interviewed by telephone, while among those under age 55, half were interviewed by telephone and half via the internet. Among Jews who were not ultra-Orthodox, most of those over the age of 65 were interviewed by telephone, while most of those under age 65 were interviewed via the internet. Up to five attempts were made to reach each sampled participant, and the response rate was 62%.

### Data Management and Analysis

The physicians study questionnaire was based on attitude questionnaires about vaccines from the literature, as well as on studies of attitudes regarding the COVID-19 issue (64, 66). The physician population was stratified according to geography, ethnicity and age.

First we conducted a pilot study among 30 physicians from different sectors to validate and formulate the questionnaire. The purpose of the pilot study was to check the wording, validate the contents, ascertain that the questions in the questionnaire were clear and contained the most common possibilities, and determine whether it was suitable to the target population. After gathering and entering the data, we implemented quality control to find errors in data entry. The quality control included checking for forms that were filled out partially and disqualifying them.

The responses of the physicians were analyzed using the statistical software SAS version 9.4. The level of significance was set at 5%. The data analysis considered two sectors (community physicians vs. hospital physicians) as well as specialization subgroups (family medicine, pediatrics, internal medicine and so on).

The background data of the physicians as well as variables such as information transparency (respondents' evaluation of information transparency, respondents' evaluation of their self-efficacy in recommendung the vaccine) were displayed using descriptive statistics: average tables and standard deviations for categorical variables and frequency tables and percentages for categorical variables.

Associations emerged between the following variables: level of self-efficacy in recommending vaccination and specialization; physicians's level of efficacy in recommending vaccination and that of physicians from other specializations; level of self-efficacy and sense of information transparency; motives for geting vaccinated and area of specialization; attitudes of responding physicians toward hesitant physicians and the system's attitude toward those physicians.

The following tests were used to examine these variables: The Kruskal-Wallis test was used to examine gaps in self-efficacy between physicians in different specializations in recommending the vaccine. Perceived self-efficacy in recommending the vaccine was examined in reference to three factors: (1) the responding physicians themselves; (2) virologists and immunologists; and (3) physicians from other areas of specialization. The Friedman test was used to compare these three self-efficacy perceptions. The Spearman correlation was used to examine the association between physicians' perceived level of self-knowledge and their ability to recommend the vaccine.

Gaps in the motives that led physicians from different specialization areas to get vaccinated were examined using Chi-Square tests for each motive separately. The Wilcoxon Signed Rank Test was used to compare physicians' attitudes toward their peers who were hesitant about or opposed to the vaccine to the position of the health system.

The public study data analysis entailed examining how socio-demographic and personal attributes are linked to the issue of physicians' knowledge. The data were analyzed using Chi Squared Automatic Interaction Detection (CHAID, also referred to as Answer Tree). This method is used to study associations between a dependent variable and a series of predictor variables (67). While the method resembles stepwise regression, it chooses the predictor with the highest significance for each level of the model. By testing the differences between groups defined by a certain independent variable, it considers the interrelations between this variable and other independent variables (68). It also allows for the use of categorical variables. The CHAID method uses F-testing when the dependent variable is an interval, t; otherwise, it uses Chi Squared testing. If the dependent variable is continuous, it divides the scale into categories based on distribution of the answers (67, 69). Due to the large number of cases in our sample, we determined that the difference between groups was significant if the level of significance was *P* ≤ 0.001. The Answer Tree method identifies the best predictors of a dependent variable out of a list of independent variables by finding the independent variable that best distinguishes those groups that are significantly different from each other regarding the dependent variable. It then accordingly divides the sample of participants into subgroups. Other than the decision of which independent variables to introduce into the analysis, no preliminary assumptions are made concerning the best distinguishing variables or the cutoff points for these variables. Both are determined by the analysis.

The first step of this stepwise analysis entails identifying the independent variable that distinguishes the groups that differ from each other with respect to the dependent variables. The participants are then divided into these subgroups. The subsequent steps involve identifying the second and third best distinguishing variables, and so on. Yet unlike the case of stepwise regression, the subsequent steps in the CHAID method do not refer to the sample as a whole but rather treat each subgroup separately. Hence, the second-best predictor is not necessarily the same for all subgroups, but rather can differ from one subgroup to another. The analysis continues until it is unable to find an additional variable that contributes to differentiating between the groups with the dependent variables.

### Ethical Considerations

The study conducted among the physicians and the study conducted among the public were approved by the Faculty of Social Welfare and Health Sciences Ethics Committee for research with human subjects at the University of Haifa (approval no. 057/21 and approval no. 088/20 accordingly).

## Results

The physicians study population included 295 physicians who worked in various sectors of the healthcare system in Israel during the COVID-19 epidemic. Table 1 describes the socio-demographic characteristics of the study population.

**Table 1:** Socio-demographic characteristics of the physician (*n* = 295).

**Characteristic**	***n*** **(%)**
Gender	
Male	153 (51.9)
Female	142 (48.1)
Age	
26–45	173 (58.6)
46+	122 (41.4)
Ethnicity	
Jewish	178 (60.3)
Arab	117 (39.7)
Workplace	
HMO	152 (51.5)
Hospital	94 (31.9)
Both hospital and HMO	36 (12.2)
Private practice	13 (4.4)
Specialization	
Family Medicine	97 (32.9)
Internal Medicine	72 (24.4)
Pediatrics	35 (11.8)
Gynecology	23 (7.8)
No specialization	23 (7.8)
Other	45 (15.2)

The physicians were asked to report their current vaccination status. Most of the physicians (81%) reported that they have already been vaccinated or intend to be vaccinated, 8% reported that they are still hesitant about getting the vaccine, 5% reported that they have already diagnosed with COVID-19, 5% reported that they refuse to be vaccinated, and 1% reported that vaccination can cause a disease flare-up for some of their patients.

Pediatricians were found to have the highest vaccination rate (85.7%), followed by internal (83.3%), gynecology (82.6%) and family physician (80.4%) (Table 2). With respect to work sector, hospital physicians have the highest vaccination rate (85.1%), followed by physicians working at HMOs and hospital (83.3%), HMOs (80.3%) and physicians working in private practice (53.8%). Furthermore, 5.1% of family physicians reported that they refused to get vaccinated, as did 23.1% of the physicians working in the private sector, 5.9% of the physicians working in the HMO, 2.8% at the HMO and hospital and 1.1% of the physicians working in the hospital sector.

**Table 2:** Physician' vaccination decisions and associations between gender, specialization and work sector (*n* = 295).

**Characteristic**		**Total**	**I was vaccinated/I plan to be vaccinated**	**I'm hesitating**	**I was diagnosed with COVID**	**I refuse to be vaccinated**	**Vaccination can cause a disease flare-up for some of my patients**
					***n*** **(%)**		
Gender	Male	153	124 (81.0)	12 (7.8)	9 (5.9)	7 (4.6)	1 (0.7)
	Female	142	115 (81.0)	13 (9.2)	7 (4.9)	7 (4.9)	0 (0.0)
Specialization	Family	97	78 (80.4)	8 (8.3)	6 (6.2)	5 (5.1)	0 (0.0)
	Internal	72	60 (83.3)	7 (9.7)	2 (2.8)	2 (2.8)	1 (1.4)
	Pediatrics	35	30 (85.7)	2 (5.7)	2 (5.7)	1 (2.9)	0 (0.0)
	Gynecology	23	19 (82.6)	3 (13.0)	1 (4.4)	0 (0.0)	0 (0.0)
	No specialty	23	18 (78.3)	0 (0.0)	3 (13.0)	2 (8.7)	0 (0.0)
	Other	45	34 (75.6)	5 (11.1)	2 (4.4)	4 (8.9)	0 (0.0)
Work sector	HMO	152	122 (80.3)	13 (8.5)	8 (5.3)	9 (5.9)	0 (0.0)
	Hospital	94	80 (85.1)	7 (7.4)	5 (5.3)	1 (1.1)	1 (1.1)
	HMO and hospital	36	30 (83.3%)	4 (11.1)	1 (2.8)	1 (2.8)	0 (0.0)
	Private medicine	13	7 (53.8%)	1 (7.7)	2 (15.4)	3 (23.1)	0 (0.0)

The physicians' reasons for vaccine hesitancy were detailed by 38% (112/295) physicians. Almost half of them (49.1%) claimed they were hesitant because the vaccine's long-term side effects had not been tested, 13.4% that the vaccine's long-term effectiveness had not been proven, 8.0% that the vaccine's effectiveness has not been sufficiently tested, 6.3% that the vaccine received emergency FDA approval rather than regular approval, 4.5% that they don't have all the information about the vaccine clinical trials, 0.9% being pregnant, 0.9% all these reasons (except pregnancy), and 17.0% for other reasons.

Physicians in this study were also asked to estimate how many of their physician colleagues expressed fears or hesitancies about being vaccinated, on a scale ranging from 1 (not at all) to 5 (very large extent). In total 27.8% of the physicians reported large (16.6%) or very large (11.2%) extent of physician colleagues expressing concerns about getting vaccinated, 30.5% reported moderate extent, and 41.7% reported small extent (30.5%) or not at all (11.2%). The average rating was 2.86 (SD = 1.16), indicating a moderate frequency of vaccination concerns.

Physicians were subsequently asked to assess how many of their colleagues who had originally expressed concerns about being vaccinated eventually got vaccinated. They were also asked to indicate what they believed were the reasons these colleagues finally were vaccinated. In total 19.7% of the physician s reported that all their hesitant colleagues were eventually vaccinated, over half (52.2%) reported that most of their hesitant colleagues were eventually vaccinated, 9.2% reported that only a few of these colleagues were vaccinated, 2.0% reported that none of these hesitant physicians were vaccinated, and 15.6% reported that they did not know whether the hesitant physicians in their area eventually were vaccinated (1.7% did not respond).

**Figure 1:**
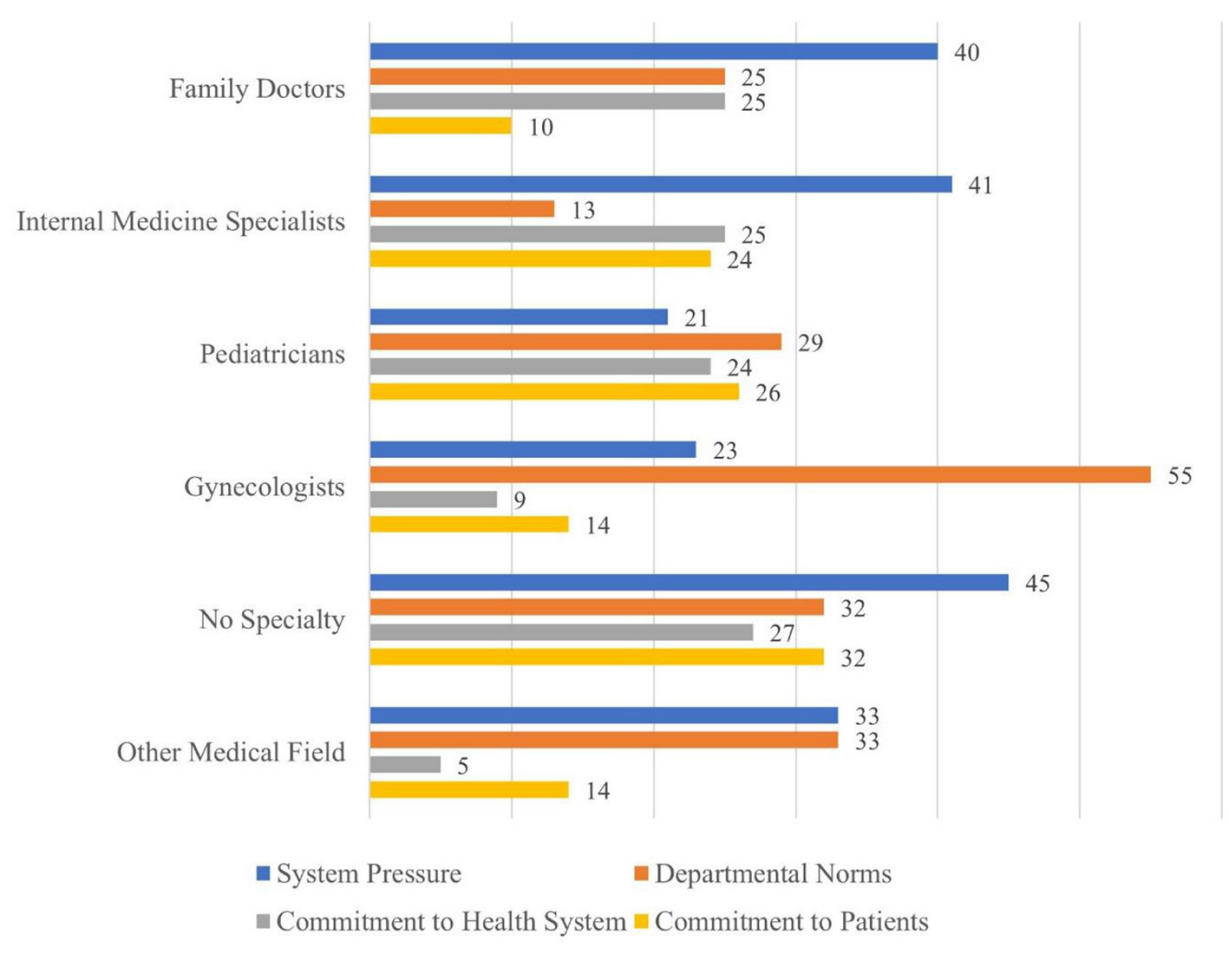
Physician's reasons for vaccination by area of specialization (%).

Figure 1 depicts the distribution of physicians' reasons for getting vaccinated by area of specialization.

Figure 1 shows that system pressure is the most vaccination reason among physicians with no specialty (45%), internal medicine specialists (41%), family physicians (40%), and physicians from other medical fields (33%). Departmental norms constitute the most vaccination reason among gynecologists (55%), pediatricians (29%) and physicians from other medical fields (33%), while on the overhaul commitment to patients is a less common reason (10%-24%).

This study focused on the issue of physicians who are hesitant or have reservations about the COVID-19 vaccine. We examined what the colleagues of these hesitant physicians think of them and how they believe the system perceives these hesitant physicians. To this end, we calculated an index to describe the system's attitude toward hesitant physicians. Using this index, we tested the correlation between the attitudes of the participating physicians and those of the health system.

The findings indicate that the health system has low tolerance for hesitant physicians (Mean = 2.33, SD = 0.93). The physicians believe that the system tends not to tolerate physicians who are hesitant about or opposed to the vaccine. In contrast, the study physicians themselves tend to be more tolerant of hesitant physicians (Mean = 3.31, SD = 1.41). Spearman's rank correlation [r(261) = −0.2445, *p* < 0.001] indicate a negative and significant correlation between physicians' own attitudes toward hesitant physicians and those of the system, such that the stronger physicians' beliefs that tolerance of hesitant physicians is justified, the lower their perceptions that the system tolerates these hesitant physicians.

The study physicians assess the transparency of information disseminated by the Ministry of Health about the COVID-19 vaccine. In total 38% of the physicians reported strong agreement (14%) or agreement (24%) with the claim that the information is fully transparent, 30% neither agree nor disagree, and 32% disagree (15%) or strongly disagree (17%). Their average response was 3.05 (SD = 1.27) on a rating scale ranging from 1 to 5, pointing to only a moderate level of consent.

The present study also sought to examine physicians' perceived level of self-knowledge and their ability to recommend and give advice to their patients regarding the COVID-19 vaccine. On the first question, physicians were asked to assess their general degree of knowledge about the vaccine on a Likert scale ranging from 1 to 5. On the second question participants were asked to indicate their degree of agreement with the following statement, on a scale from 1 to 5: “I do not currently have sufficient tools to recommend the COVID-19 vaccine”. The third question sought to assess the extent of participants' agreement with the following statement: “I feel I do not have enough information to judge the current debate over the effectiveness and safety of the vaccine”.

During the statistical analysis, responses to these three questions were gathered into a single index. This index revealed that participants' average knowledge and self-efficacy was 3.37 on a scale from 1 to 5 (Cronbach's alpha 0.74), pointing to an above average level of perceived knowledge and efficacy.

To examine whether area of specialization is related to perceived ability to recommend the COVID-19 vaccination, we examined mean perceived recommendation ability on a scale ranging from 1 to 5, where 1 indicates complete inability and 5 indicates complete ability. Three different perceptions were examined. The first refers the study physicians' own perceived self-efficacy in recommending the vaccine, the second refers to their perceptions of the ability of physicians specializing in infectious diseases and immunologists to recommend the vaccine, and the third refers to the study physicians' perceptions of the ability of physicians in other areas of specialization to recommend the vaccine. Subsequently, Friedman's testing was used to compare these three perceptions: physicians' own perceived self-efficacy, ability of infectious disease physicians or immunologists, and ability of physicians from other areas.

Among the study physicians (none of whom specialized in infectious diseases), the mean score for self-efficacy in recommending the COVID-19 vaccination was 3.51 out of 5 (SD = 1.27), pointing to an above average level of self-efficacy. The study physicians' average scores for perceived ability of infectious disease physicians (Mean = 3.50, SD = 1.28) and perceived ability of physicians from other areas (Mean = 3.49, SD = 1.20) to recommend the vaccination were similar. The result of Friedman's testing to compare these three perceptions was not significant [χ^2^(2) = 1.1489, *P* = 0.5630]. Hence, we can conclude that the study physicians' assessments of their own ability to recommend the vaccine do not differ from their assessments of other physicians' recommendation abilities, regardless of whether or not the physicians specialize in infectious diseases.

Spearman's rank correlation [r(295) = 0.5159, *p* < 0.0001] indicate positive correlation between perceived knowledge about the vaccine and perceived self-efficacy in recommending the vaccine among the study physicians.

The study physicians' opinions regarding government measures taken to control the pandemic indicate that most (71%) of the physicians think that the steps taken by the government are erroneous (54%), excessive (14%), or even unnecessary (3%), while only 29% think these measures are necessary. The study physicians' attitudes toward the government's strategy in dealing with the COVID-19 pandemic indicate that most physicians (75%) indicated that the government is pursuing a strategy of fear appeal, 15% a strategy of mutual responsibility, 12% of support, and 8% of transparency.

**Figure 2:**
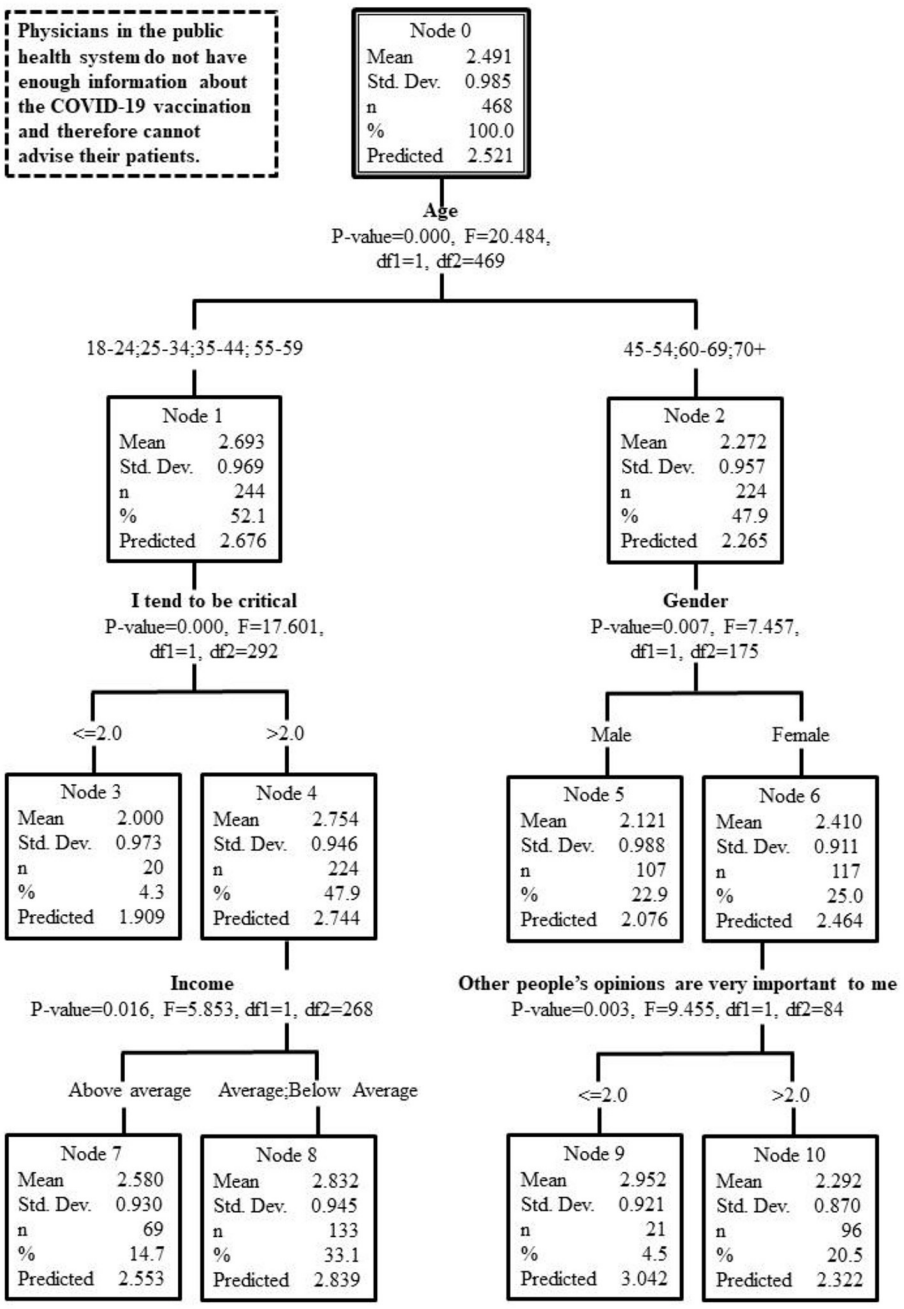
Chart showing results of the CHAID analysis conducted among the public.

The results of the CHAID analysis conducted among the public are depicted in Figure 2.

As the figure shows, among the public, the main explanatory variable was age. People up to the age of 44 and people between the age of 55–59, agree with the contention that physicians today do not have sufficient information about the COVID-19 vaccine. On a four-point scale the average for people up to the age of 44 and people between the age of 55–59 was 2.69, compared to 2.27 among people between the age of 45–54 and above the age of 60 (*P-*value = 0.000, F = 20.484, df1 = 1, df2 = 469).

Another explanatory variable among people up to the age of 44 and people between the age of 55–59 was the personal attribute of being critical. Those who tend to be critical believe that physicians do not have sufficient information (average of 2.75 on a four-point scale), compared to an average of 2.00 among those who are generally not critical (*P*-value = 0.000, F = 17.601, df1 = 1, df2 = 292). Among critical people, another explanatory variable is income, such that those with an average or below average income have a greater tendency to think that physicians do not have enough information (2.83), compared with those who have an above average income (2.58) (*P*-value = 0.016, F = 5.853, df1 = 1, df2 = 268).

Among people between the age of 45–54 and above the age of 60, gender is also an explanatory variable. Women believe that physicians do not have enough information (2.41), compared to men (2.12) (*P*-value = 0.007, F = 7.457, df1 = 1, df2 = 175). Among women, the factor of caring what others think also serves as an explanatory variable, such that those who do not care what others think have stronger beliefs that physicians do not have enough information (2.95) than those who care what other people think (2.29) (*P*-value = 0.003, F = 9.455, df1 = 1, df2 = 84).

## Discussion

The study found that most of the study physicians (81%) reported they had already been vaccinated or intended to be vaccinated. An examination of the physicians by sector shows that 5.1% of the family physicians working in the community reported they refused to be vaccinated, as did 23.1% of the physicians working in private practice. These percentages are high relative to other sectors.

One possible explanation for this finding is that because the coronavirus pandemic has been labeled an emergency situation, most COVID-19 patients do not seek treatment from physicians working in the community. Rather, when their condition deteriorates, they go to the hospital for treatment. Hence, hospitals are stricter about vaccinating the medical staff. Another explanation is that physicians in the community are more independent in making decisions and have less direct contact with their superiors. This point also apparently explains the findings for physicians in the private sector, who are more independent than those working in other sectors and freer to make their own decisions.

As noted, most of the study physicians indicated they had already been vaccinated or intended to be vaccinated. Yet when asked about their reasons for vaccine hesitancy, 33% cited concerns about long-term side effects (24%) along with doubts about the vaccine's effectiveness in preventing contagion (3%) and other reasons (6%).

Other studies that examined physicians during the COVID-19 pandemic yielded similar results. For example, a study conducted in the US at the end of 2020 examined attitudes and behavioral intentions regarding the COVID-19 vaccine among HCWs in advance of the vaccination campaign (70). The results showed that 36% of the respondents expressed their willingness to be vaccinated as soon as the vaccine became available, while 56% were unsure and were waiting for additional information. The most prevalent concerns regarding the COVID-19 vaccine were safety (69%), effectiveness (69%) and the vaccine's rapid development/approval process (74%).

These concerns expressed by HCWs resemble those found in studies examining HCWs' hesitancy to get influenza vaccinations (10, 11). Studies of HCWs have found that their vaccination barriers include concerns about side effects, vaccination novelty, and lack of faith in the vaccine's efficacy and in the severity of the disease (10, 11, 13).

A significant percentage of the physicians in the current study (27.8%) reported seeing vaccine hesitancy among their immediate colleagues. When these physicians were asked if they knew whether these hesitant colleagues eventually were vaccinated, they reported that 71.9% of all of them (19.7%) or most of them (52.2%) did get vaccinated.

The physicians cited two reasons for this eventual compliance: pressure from the system and departmental norms. These two reasons in effect do not derive from perceived risk of the coronavirus disease but rather are external reasons stemming from interaction with the system.

Gynecologists noted that departmental norms (55%) played a major role in their decision to be vaccinated, as opposed to their obligations toward their patients (14%). This finding is ostensibly surprising in that one would expect that gynecologists who work with pregnant women and new mothers on a daily basis are concerned about safeguarding mothers and newborns. Yet it is possible that gynecologists do not perceive the vaccination in terms of protection against infection and are therefore not motivated to be vaccinated by risk perceptions but rather by departmental norms. Similarly, we found that among family physicians the main reason for getting vaccinated is system pressure, rather than their sense of obligation to their patients, which was only 10%. Hence, we hypothesize that, like gynecologists, family physicians did not see the vaccine as a way of protecting their patients, such that their main motivation to be vaccinated was pressure from the system.

System pressure on HCWs to get vaccinated has been a longstanding strategy, particularly during epidemiological crises. A “solution” for reducing the gap between declarations and actual behavior that is often heard at the organizational level is to “crack down” by compelling HCWs to get vaccinated (7). This coercion can be in the form of legislation or by means of punishments meted out by the system. Yet turning a voluntary act into something that is mandatory raises obvious ethical and legal issues (71–73). Nevertheless, the idea of forcing medical personnel to get vaccinated often resurfaces in internal discussions in government ministries or is reflected by authority or force exercised by governmental bodies that push hospital directors to pressure their staff. For example, in 2009 the state of New York issued a seasonal influenza vaccination mandate for healthcare workers (74). In Israel in 2014, the administrations of several hospitals decided to require physicians and nurses who received influenza vaccinations to wear tags indicating they had been vaccinated (72). In October 2015, the Israel Health Ministry instructed the management of hospitals and HMOs to require all medical personnel to be vaccinated against influenza (75).

According to social marketing (76) and risk communications (77) approaches, in order for any change to be internalized, government authorities must engage in shared dialogue that is not paternalistic with all relevant target population groups. In this case, what is required is engaging in dialogue with HCWs who represent the health system. The literature on risk communications indicates that risk perception entails not only the scientific risk but also the worries and concerns of those involved (78, 79). Sandman (80) contends that risk perception is composed of hazard plus outrage. That is to say, other than scientific aspects, feelings of outrage toward the risk must also be considered. Correspondingly, lack of agreement between authorities and physicians' perceptions of hazard and outrage can lead to controversy (80).

This controversy cannot be eliminated by coercion or by exercising authority, particularly during situations of uncertainty such as a pandemic. Uncertainty/ambiguity is often associated with decreased willingness to adopt preventive measures such as vaccination (81). Indeed, research shows that access to honest and diversified information can encourage participation in decision-making about health risks (82, 83).

In line with this research, rather than forcing physicians to comply, health authorities should provide physicians with transparent information and involve them in the process of making decisions regarding the COVID-19 vaccination. Moreover, the findings of the current study indicate that physicians may be in a state of cognitive dissonance (84), such that their professional obligation to recommend vaccination to their patients clashes with their personal values and perceptions. The literature rarely deals with the factors underlying this ambivalence and or with the barriers and concerns that can negate or undermine physicians' professional attitudes (85). This dissonance is often reflected in a gap between the declarative level—i.e., HCWs' recommendations to patients to get vaccinated—and the behavioral level, as manifested in their own reluctance to be vaccinated with the very same vaccines they recommended.

The findings of the current study indicate that the health system tends not to tolerate physicians who are hesitant about the COVID-19 vaccination or who actually oppose it, whereas the study physicians tend to be more tolerant. This result leads to the conclusion that the point of view of the system does not reflect the views of the physicians themselves. Indeed, it appears that the study physicians are more understanding of vaccine hesitancy and of the reasons for being unsure about the vaccine. One of the reasons for this greater tolerance may be that even physicians who were vaccinated had concerns and doubts about the COVID-19 vaccine. Therefore, they are able to identify and empathize with hesitant physicians, despite their own willingness to be vaccinated.

Moreover, scientists and people in the medical profession are encouraged to ask questions, such that expressing doubts is an integral part of the medical profession. Therefore, physicians tend to be more tolerant of skeptical views.

These findings are in line with findings that during the COVID-19 crisis health systems across the globe, and in Israel as well, have been impatient with professional criticism directed at them. One example of this is the medical establishment's disregard and criticism of the Barrington declaration (86, 87), which opposed a policy of lockdowns and tests for people without symptoms.

Indeed, the medical establishment worldwide has been attacking physicians who have come out against the vaccination policy, labeling them as spreaders of fake news (88) and disregarding their views. A case in point is the Rome Declaration (89), which attracted more than 10,700 signatures from physicians worldwide. The declaration calls for a halt to the reckless use of Dr. Malone's invention—mRNA platform technology—and renewed attention to human rights.

In a study that examined the scientific discourse in Israel at the beginning of the COVID-19 pandemic, we found that there was no dialogue between opposition and coalition experts. Moreover, the coalition experts labeled the experts who criticized them as “coronavirus deniers” and “anti-vaxxers” (62). During the vaccination campaign in Israel, the health system rejected criticism from opposition experts in several ways: It refused to engage in discourse with opposition experts (90). The Ministry of Health's Committee for the Prevention of Misleading the Public in Advertising sent letters of reproach to physicians who reported on or issued warnings about the vaccine's side effects (91), while those under attack submitted claims against the Ministry of Health (92). Moreover, as noted, those critical of Ministry of Health policy were called names and labeled as “anti-vaxxers” (93).

The health establishment's tendency to silence opposing voices has several explanations, some deriving from the perception (94) that scientific consensus is the only way to construct scientific policy. One motivation is the desire to block doubters with economic and political interests who try to sow confusion and fear in the minds of the public (95). Moreover, the majority is often concerned that a minority opinion will impede its ability to reach group consensus and convey a coherent message.

When the physicians of the current study were asked to assess the transparency with which the health establishment conveyed information about the COVID-19 vaccine at the beginning of the campaign, they rated government transparency as moderate rather than high. Moreover, 32% of the physicians did not believe that the information conveyed to them was fully transparent, while 30% did not express an opinion about information transparency, representing a considerable proportion of the study physicians. This may reflect the fact that at the beginning of the vaccination campaign, the physicians themselves did not yet know what information they should be receiving.

Moreover, the study physicians rated their own knowledge as average. Hence, their perceived knowledge mirrored their perception of how the system conveyed this knowledge. In addition, most of the study physicians identified intimidation as the government's primary strategy. This perception is in line with the findings of a rhetorical analysis of the media strategy adopted by the government of Israel during the COVID-19 crisis (96). It also reflects the public's views of the government's policy (97, 98). Indeed, intimidation has been a major strategy, along with other means such as comparisons to historical epidemics with many casualties, frightening predictions of morbidity and mortality rates, public accusations and more (96, 98).

The findings of the current study reveal a correlation between level of knowledge and level of self-efficacy. That is, as physicians acquire more knowledge, their self-efficacy in recommending the vaccine also increases. This leads to the conclusion that it is very important for physicians to feel that their level of knowledge is high rather than average (as indicated by most of the study physicians) in order to reinforce their self-efficacy.

When the study physicians were asked whether the self-efficacy of epidemiologists and virologists differed from that of other physicians, they answered that there were no differences. This finding is ostensibly surprising. One would expect that physicians who are not in this field would feel less confident about recommending the vaccination than their colleagues specializing in this field. One possible explanation for this finding is that because the COVID-19 vaccine was created using a new technology, the study physicians thought that epidemiologists and virologists probably did not know more about the vaccine than they did. Another possible explanation is that the health system gave family physicians and gynecologists equal authority in recommending the vaccine to their patients, leading these physicians to think they were not lacking in knowledge. In so doing, the system “flattened” the requirements for recommending the vaccine. That is, the system considers all physicians to be their agents in conveying the message and does not expect them to have in-depth knowledge about vaccinations. In other words, all physicians are expected to tell their patients of the advantages of the vaccine while limiting discussions of the vaccine's components or the resulting immunological processes.

At the beginning of the vaccination campaign, the public was asked whether physicians had sufficient information about the vaccine. The results show that mostly young people up to the age of 44 agree with the contention that physicians do not have enough knowledge. Another explanatory variable among young people is a personal tendency to be critical. Those who are critical are more likely to think that physicians do not have enough knowledge than those who are not critical. This finding is in line with the findings of another study we conducted during the COVID-19 pandemic in Israel. Among other things, that study examined the influence of age and tendency to be critical on support for the medical establishment in handling the crisis. The study found that people who tend toward conservatism also tend to support the establishment's views and to accept its approach and claims, as opposed to those who tend to be less conservative. Each of these groups has an additional explanatory variable. Among those who are conservative, the additional explanatory variable is age, such that those age 45 and above show more support for the government, while those age 44 and younger give more support to the opposition's positions (62).

In addition, the findings of the current study are in line with studies showing that young people are less established and tend to be less conservative (99, 100) than older people and to adopt anti-establishment opinions and approaches in politics, as well as in health policies.

Among older adults, gender serves as an additional explanatory variable. Older women are more likely than older men to think that physicians do not have sufficient knowledge. One explanation for this finding is that women consume more health services and are more involved in making decisions about their health and the health of their spouses (101). Hence, they also read more and are more exposed to critical opinions than are older men.

### Study Limitations

One limitation of this study is related to the sampling method, which may entail selection distortion in that the study physicians were recruited via social networks and snowball sampling. Nevertheless, the study population comprised physicians who are currently coping with a crisis. Even during routine times reaching this population is difficult, making this recruitment method the most effective method. In addition, the study was conducted at the beginning of the COVID-19 vaccination campaign in Israel. Further research is needed to examine changes in physicians' viewpoints and perceived self-efficacy. We recommend conducting mixed studies that use both quantitative and qualitative methods in order to examine the interpretations together with the physicians themselves.

## Conclusion

The findings of this study indicate that the health system should employ complete transparency in conveying the advantages and disadvantages of the COVID-19 vaccine to physicians. The health system should be more tolerant of physicians' worries and concerns and should give them the sense that their reservations and misgivings are legitimate. Moreover, medical studies should reinforce physicians' immunological knowledge regarding vaccinations so they can help their patients make informed decisions in the given context.

## Data Availability Statement

The raw data supporting the conclusions of this article will be made available by the authors, without undue reservation.

## Ethics Statement

The studies involving human participants were reviewed and approved by Faculty of Social Welfare and Health Sciences Ethics Committee for research with human subjects at the University of Haifa. The patients/participants provided their written informed consent to participate in this study.

## Author Contributions

AG-E has supervised this study that was carried out by HK as part of her MHA thesis and wrote the first draft. HB-K collected the data. AG-E and HB-K analyzed the data. All authors reviewed and modified the article. All authors contributed to the article and approved the submitted version.

## Conflict of Interest

The authors declare that the research was conducted in the absence of any commercial or financial relationships that could be construed as a potential conflict of interest.

## Publisher's Note

All claims expressed in this article are solely those of the authors and do not necessarily represent those of their affiliated organizations, or those of the publisher, the editors and the reviewers. Any product that may be evaluated in this article, or claim that may be made by its manufacturer, is not guaranteed or endorsed by the publisher.
