# Supplementary material for: Physicians' Perspective on Vaccine-Hesitancy at the Beginning of Israel's COVID-19 Vaccination Campaign and Public's Perceptions of Physicians' Knowledge When Recommending the Vaccine to Their Patients: A Cross-Sectional Study
Source: Front Public Health. 2022 Mar 10;10:855468. doi: 10.3389/fpubh.2022.855468 (PMC8960033; doi:10.3389/fpubh.2022.855468)
Supplement: Supplementary file 1 [file Data_Sheet_1.PDF]

frontiers

Supplementary Material

Questionnaire examining socio-demographic and personal attributes

1. Please indicate the extent to which each of the following describes you, on a scale from 1 to 7, where 1 means “does not describe me at all” and 7 means “describes me completely”:

	1	2	3	4	5	6	7	Don’t know
1. I am critical								
2. I care about other people’s opinions								
3. I am anxious								
4. I am open to new experiences								
5. I am conservative/ I follow the norms								
6. I tend to take responsibility even when I know others are responsible for a particular matter								

2. How old are you? ___________

3. The average gross household income in Israel is NIS 13,500 (NIS 7,950 gross for individuals living alone). Rate your household’s income relative to the average on the following rating scale:

1. Well below average	2. A bit below average	3. Average	4. A bit above average	5. Well above average	9. Decline to answer

4. Indicate your highest level of education on the following rating scale:

1. Elementary/some high school	2. High school	3. Tertiary education	4. Academic	9. Decline to answer

5. Indicate whether you have any underlying conditions (e.g., diabetes, oncology patient, heart condition, and so forth) on the following rating scale:

1. I have an underlying condition	2. I do not have any underlying conditions	9. Decline to answer

6. Where were you born? Indicate on the following rating scale:

1. Israel	2. Former Soviet Union	3. Other	9. Decline to answer

7. Did you immigrate before or after 1990? Indicate on the following rating scale:

1. Before 1990	2. 1990 and later	9. Refuse to answer

8. Marital status. Indicate on the following rating scale:

1. Married/ in a relationship/ divorced /widowed, with children	2. Married/in a relationship/ divorced /widowed, no children	3. Single, not in a relationship, with children	4. Single, not in a relationship. no children	9. Decline to answer

9. Based on your family's ethnic origin, how would you define yourself on the following rating scale:

1. Ashkenazi	2. Mizrahi	3. Sephardi	4. Combined Ashkenazi/ Mizrahi	5. Native of Former Soviet Union	6. Member of Ethiopian community	7. Other	9. Decline to answer
